# Anomalous Coronary Origin and Malignant Course in a Young Patient With Palpitation and Demand Ischemia: A Serendipity or a Syndrome Without a Name?

**DOI:** 10.7759/cureus.13491

**Published:** 2021-02-22

**Authors:** Udit Joshi, Harsh Rawal, Varun Vanka, Sudhir Mungee

**Affiliations:** 1 Cardiovascular Medicine, University of Illinois College of Medicine, Order of St. Francis Medical Centre, Peoria, USA; 2 Internal Medicine, Advocate Illinois Masonic Medical Center, Chicago, USA; 3 Internal Medicine, University of Illinois College of Medicine at Peoria, Peoria, USA; 4 Cardiology, University of Illinois College of Medicine, Order of St. Francis Medical Centre, Peoria, USA

**Keywords:** anomalous rca, palpitations, coronary artery, coronary artery angiography

## Abstract

Anomalous coronary artery remains the second most common cause for sudden cardiac death (SCD) in young athletes. The anomaly most commonly associated with SCD is the one that courses between the aorta and pulmonary artery, the malignant course. We present a case of a young gentleman who presented with symptomatic palpitations and was found to have anomalous right coronary artery from ostial left main coronary artery coursing between the aorta and pulmonary artery.

## Introduction

Troponin elevation is a marker of myocardial injury, and when associated with atypical symptoms, it is always challenging to gauge the extent of workup. It gets more complex when there is a patient with the lowest pretest probability for coronary artery disease and otherwise has an alternate etiology for troponin elevation​​​​.* *Not to be forgotten, acute coronary syndrome is not the only reason for troponin elevation and particularly in young patients, when there are other pathologies possible which, if not actively looked for, can be lethal. One such finding is the coronary artery's anomalous origin, which is the second most common cause of sudden cardiac death in young patients. Here, we present a case of a young man with minimal troponin elevation, and atypical symptoms who had an anomalous right coronary artery originating from the ostial left main with the inter-arterial course.

## Case presentation

A 33-year-old man with a history of hypertension, obesity, and five pack-year smoking history presented to the hospital for symptomatic palpitations. He had a short sprint with his friend, following which the palpitations didn't resolve for almost three hours. He checked on a couple of heart monitors at home, which showed his heart rate in the range of 180-190s. His symptoms constituted shortness of breath and diaphoresis. Prior to the presentation, he was very active and never had similar complaints in the past. There was no history of drug use, and his urine drug screen was negative.

On arrival to the emergency department, his symptoms were completely resolved. Electrocardiogram (EKG) showed normal sinus rhythm with no ischemic ST-T changes. As a part of workup, troponin up-trended reaching peak at 0.7 (normal: <0.030; trend: 0.01-->0.14-->0.7-->0.5). Considering sudden onset symptomatic tachyarrhythmia, he got a CT angiogram chest, which ruled out pulmonary embolism and normal thyroid-stimulating hormone (TSH) levels. Transthoracic echocardiogram showed a normal ejection fraction of 65% with moderate left ventricular hypertrophy. There were no regional wall motion abnormalities. He was hypertensive on presentation with a blood pressure of 190/100 mmHg. Ideally, his troponin elevation could have been explained by hypertension and presumed tachyarrhythmia; however, it was important to rule out acute coronary syndrome from premature coronary artery disease or myocarditis. Hence, he was started on management for acute coronary syndrome and scheduled for a left heart catheterization. 

**Figure 1:**
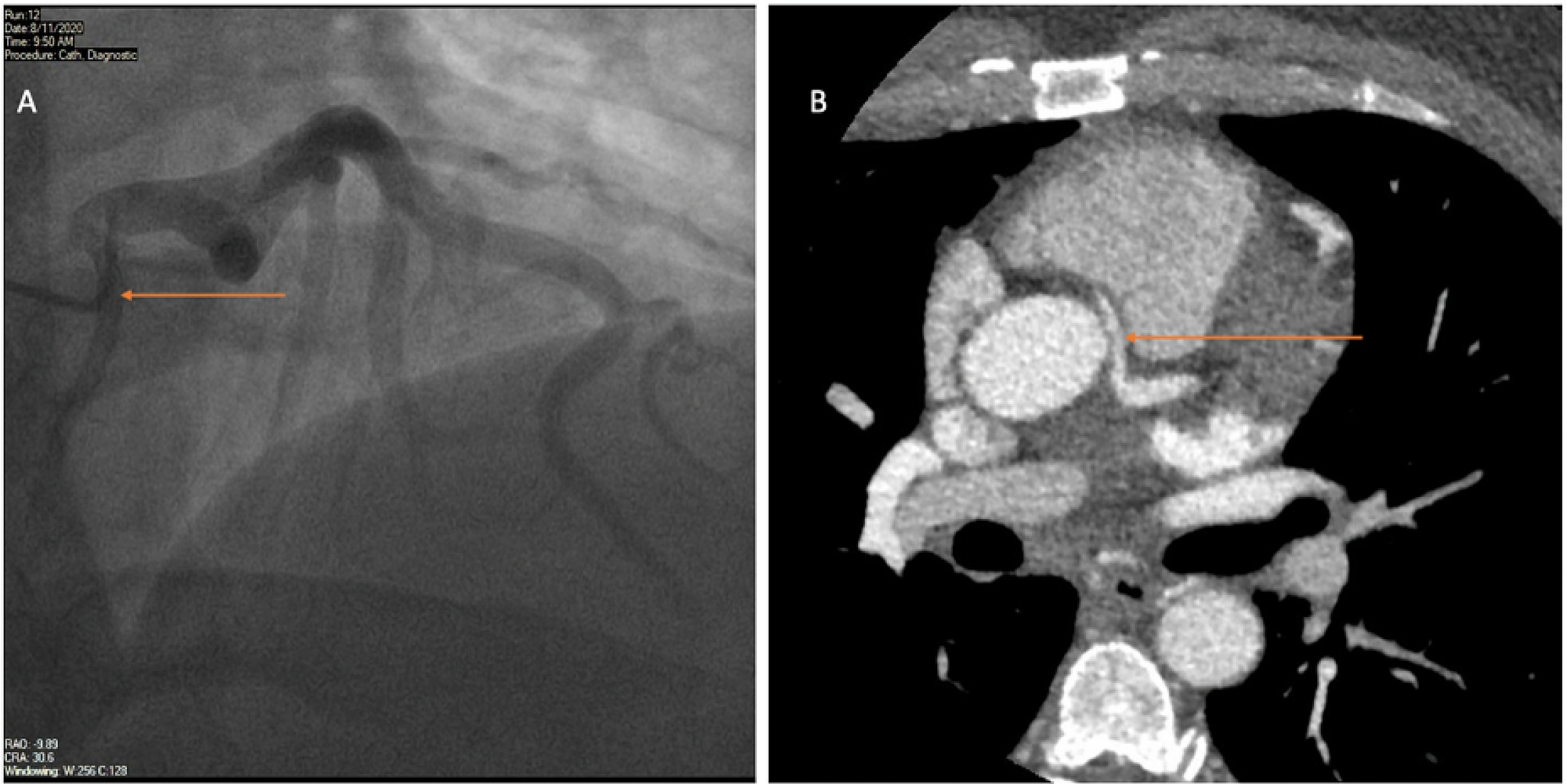
Coronary and CT angiograms A: Coronary angiogram with right anterior oblique (RAO) cranial view showing right coronary artery offsetting from the left main coronary artery ostium. B: Computed tomography (CT) angiogram showing the course of the anomalous right coronary artery between the aorta and pulmonary artery.

He underwent left heart catheterization, and to our surprise, he was found to have an anomalous origin of the right coronary artery from the ostial left main coronary artery with a codominant system. No significant stenosis or culprit lesions were found (Figure 1).

**Figure 2:**
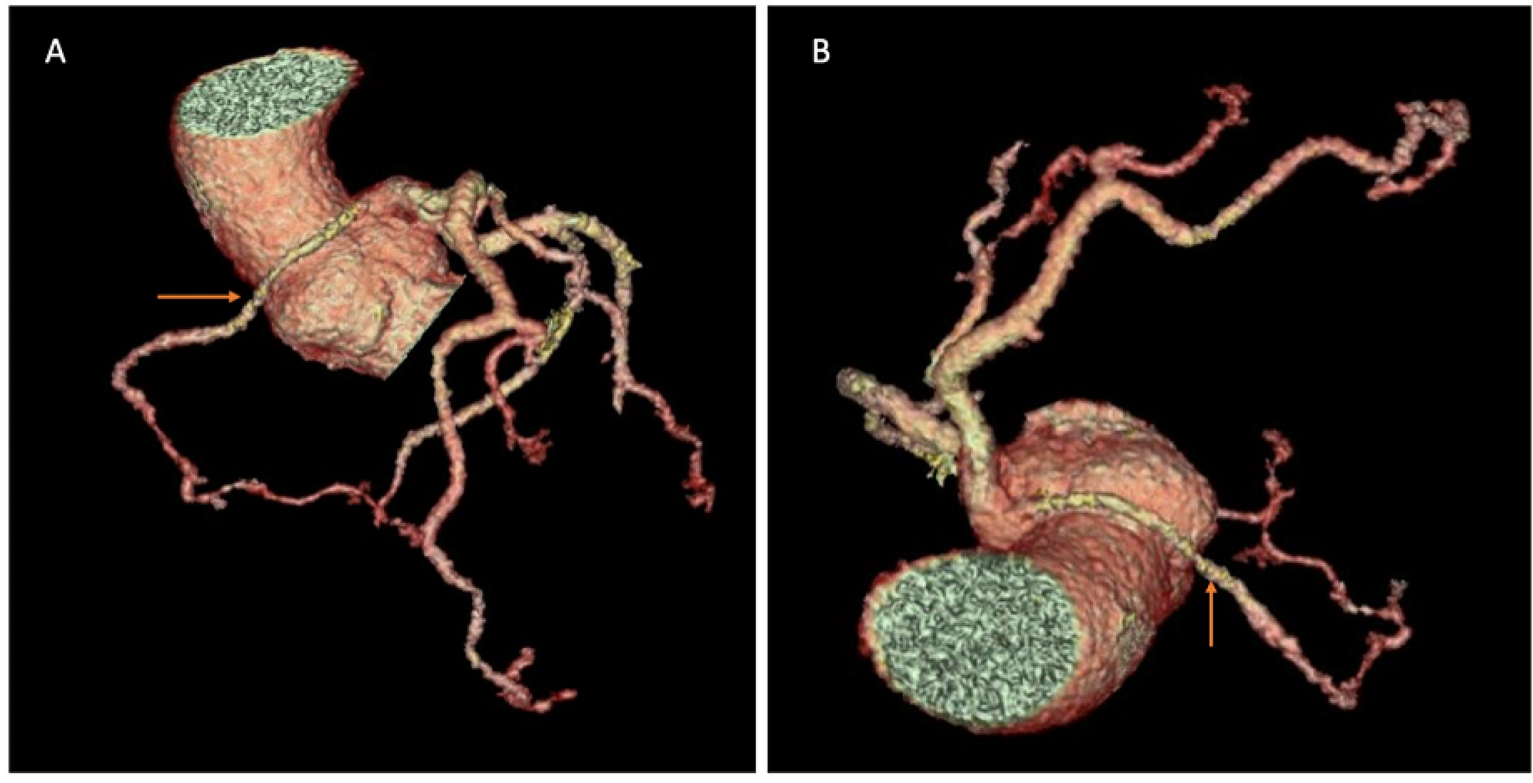
Three-dimensional reconstruction A, B: 3-dimensional reconstruction showing the anomalous course of the right coronary artery that is originating from the left main coronary artery ostium.

He underwent coronary CT angiography (CTA) to better define the course of coronary arteries, which confirmed the anomalous origin and revealed an inter-arterial course between the aorta and main pulmonary artery. Also, the proximal segment of the right coronary artery (RCA) had a slightly flattened appearance as it coursed between the great vessels. The anomalous course and origin were reconfirmed on 3-dimensional reconstruction on different views (Figure 2).

Considering this malignant course of the co-dominant RCA, it had a high risk of ventricular arrhythmia and sudden cardiac death. Hence, cardiothoracic surgery was consulted and underwent re-implantation of an anomalous right coronary artery using an internal mammary artery on the anterior aspect of the aorta.

The hospital course was uneventful, and the patient was discharged on the fourth day. Three weeks after discharge, he was followed up in the office and was recovering well without any recurrence of symptoms.

## Discussion

In patients undergoing coronary angiography, the incidence of coronary anomalies is around 0.3-1% [1, 2]. Malignant RCA anomaly is extremely rare, with an incidence of 0.03-0.17% [3]. Coronary anomalies are the second most common cause of sudden cardiac death (SCD) during strenuous physical activity in young athletes after hypertrophic cardiomyopathies, accounting for 19% [4, 5]. In a retrospective analysis involving 12,457 patients undergoing coronary angiogram, there were no cases reported for RCA originating from the left main coronary artery (LMCA). There are only a few isolated case reports with such coronary anomaly, and their presentation is usually dramatic, such a sudden cardiac death, ST-segment elevation myocardial infarction (STEMI), or ventricular arrhythmias [1, 4, 6]. The true incidence is likely underestimated as coronary angiography is not performed until the patient is symptomatic.

Coronary anomalies can be categorized based on their origin and course. Three subtypes of anomalous RCA courses have been described: 1. high inter-arterial course, between the aorta and pulmonary artery; 2. hypoplastic anomalous RCA orifice; and 3. a low inter-arterial course that runs between the aorta and right ventricular outflow tract [7]. The timing of symptoms is unpredictable, and the first symptom could itself be sudden cardiac death. Symptomatology, diagnosis, and treatment vary with the type of anomaly. The symptoms could range from exertional anginal chest pain and transient palpitations to ventricular arrhythmias, acute myocardial infarction, and SCD. Rarely, RCA originating from the left coronary cusp can cause heart blocks; and the one originating from the pulmonary artery can range from asymptomatic to varied presentation of myocardial ischemia [8, 9].

The exact mechanism of arrhythmias and SCD is not clear however various hypothesis has been proposed. As these events are usually seen in young athletes with the malignant course during exertion, it is presumed that exercise causes dilatation of the aortic and pulmonary trunk, which may further increase the angulation of the anomalous artery leading to slit-like orifice diameter and myocardial ischemia. Other potential mechanisms include spasm of the anomalous artery, endothelial injury, or ischemia caused by its long course and anomalous course predisposes to premature atherosclerosis [10].

Routine testing with rest or stress EKG is not diagnostic of such anomaly, and it does not help to predict the risk of SCD. CT coronary angiogram or percutaneous coronary angiography are the most reliable imaging modalities for diagnosis. The usual modality of treatment in symptomatic patients is almost always surgical. The commonly preferred treatment methods are unroofing procedure, bypass grafts, or reimplantation of the anomalous artery to its appropriate coronary sinus [9]. Indications for surgery patients with anomalous RCA include ischemic symptoms, ischemia on the stress test, arrhythmias, or malignant course. In contrast, almost all patients with the anomalous left coronary artery (LCA) have indications for corrective surgery [11].

## Conclusions

Our patient did have atypical symptoms along with troponin elevation, and it was likely secondary to tachyarrhythmia and elevated blood pressure on presentation; however, it was prudent to rule out acute coronary syndrome and myocarditis. He received left heart catheterization the next day and discovered a malignant course of anomalous RCA, which otherwise could have been life-threatening if undiagnosed. Tachycardia and elevated blood pressure probably lead to expansion of the aortic and pulmonary trunk, compressing the RCA leading to anginal equivalent symptoms. RCA originating from ostial left main along with malignant course is extremely rare. Hence, it is very important to look for definitive answers rather than presumption if there is any uncertainty with the case as it may be lifesaving.
